# A Co-Opted DEAD-Box RNA Helicase Enhances Tombusvirus Plus-Strand Synthesis

**DOI:** 10.1371/journal.ppat.1002537

**Published:** 2012-02-16

**Authors:** Nikolay Kovalev, Judit Pogany, Peter D. Nagy

**Affiliations:** Department of Plant Pathology, University of Kentucky, Lexington, Kentucky, United States of America; University of California Riverside, United States of America

## Abstract

Replication of plus-strand RNA viruses depends on recruited host factors that aid several critical steps during replication. In this paper, we show that an essential translation factor, Ded1p DEAD-box RNA helicase of yeast, directly affects replication of *Tomato bushy stunt virus* (TBSV). To separate the role of Ded1p in viral protein translation from its putative replication function, we utilized a cell-free TBSV replication assay and recombinant Ded1p. The *in vitro* data show that Ded1p plays a role in enhancing plus-strand synthesis by the viral replicase. We also find that Ded1p is a component of the tombusvirus replicase complex and Ded1p binds to the 3′-end of the viral minus-stranded RNA. The data obtained with wt and ATPase deficient Ded1p mutants support the model that Ded1p unwinds local structures at the 3′-end of the TBSV (−)RNA, rendering the RNA compatible for initiation of (+)-strand synthesis. Interestingly, we find that Ded1p and glyceraldehyde-3-phosphate dehydrogenase (GAPDH), which is another host factor for TBSV, play non-overlapping functions to enhance (+)-strand synthesis. Altogether, the two host factors enhance TBSV replication synergistically by interacting with the viral (−)RNA and the replication proteins. In addition, we have developed an *in vitro* assay for Flock house virus (FHV), a small RNA virus of insects, that also demonstrated positive effect on FHV replicase activity by the added Ded1p helicase. Thus, two small RNA viruses, which do not code for their own helicases, seems to recruit a host RNA helicase to aid their replication in infected cells.

## Author Summary

Subverted host factors play a role in plus-strand RNA virus replication. Small RNA viruses do not code for their own helicases and they might recruit host RNA helicases to aid their replication in infected cells. In this paper, the authors show that the Ded1p DEAD-box helicase, which is an essential translation factor in yeast, is recruited by *Tomato bushy stunt virus* (TBSV) into its replicase complex. They also show that Ded1p binds to the viral (−)RNA and promotes (+)-strand TBSV synthesis when added to a yeast-based cell-free extract depleted for Ded1p. An ATPase defective Ded1p mutant failed to promote TBSV replication *in vitro*, suggesting that the helicase activity of Ded1p is essential for its function during TBSV replication. In addition, the authors also show that another host protein, which also binds to the (−)RNA, namely glyceraldehyde-3-phosphate dehydrogenase (GAPDH), further enhances TBSV (+)RNA when added together with Ded1p to yeast-based cell-free extract. In summary, the authors show that the major functions of Ded1p and GAPDH host proteins are to promote TBSV replication via selectively enhancing (+)-strand synthesis.

## Introduction

All eukaryotic plus-stranded (+)RNA viruses have similar replication cycles in infected cells. After translation of their mRNA-sense genomic RNA(s), the viral RNA and the viral replication proteins are recruited to the site of viral replication in membranous compartments. After the assembly of the membrane-bound viral replicase complexes (VRC), the viral replicase uses the viral RNA as a template to produce complementary (−)RNA. This is then followed by (+)-strand synthesis in an asymmetric manner, producing excess amounts of (+)-strand progeny, which is released from replication for other viral processes. For efficient replication, (+)RNA viruses recruit numerous host proteins [1]–[5]. Among the identified host proteins are RNA-binding proteins, such as translation factors, ribosomal proteins and RNA-modifying enzymes [1]. The co-opted host proteins likely affect several steps in viral RNA replication, including the assembly of the replicase complex and/or viral RNA synthesis. However, the functions of host factors in (+)RNA virus replication are known only for a small number of host factors [1]–[9].

*Tomato bushy stunt virus* (TBSV) is a model plant RNA virus that is used intensively for identification and characterization of host factors [1], [10]–[12]. The single genomic RNA codes for two replication proteins, p33 and p92^pol^, which are sufficient to support TBSV replicon (rep)RNA replication in yeast (*Saccharomyces cerevisiae*) model host [13], [14]. p33 and p92^pol^ are components of the membrane-bound VRC that also requires the tombusviral (+)repRNA as a platform during its assembly and activation [11], [15]–[17]. Recent genome-wide screens and global proteomics approaches with TBSV based on yeast revealed a large number of host factors interacting with viral components or affecting TBSV replication. A large fraction of the identified host proteins are RNA binding proteins, which might affect viral RNA synthesis [10], [18]–[20].

The highly purified tombusvirus VRC is known to contain at least six permanent resident host proteins, including the heat shock protein 70 chaperones (Hsp70, Ssa1/2p in yeast) [21]–[24], glyceraldehyde-3-phosphate dehydrogenase (GAPDH, encoded by *TDH2* and *TDH3* in yeast) [25], pyruvate decarboxylase (Pdc1p) [24], Cdc34p E2 ubiquitin conjugating enzyme [24]–[26], eukaryotic translation elongation factor 1A (eEF1A) [27], [28], eukaryotic translation elongation factor 1Bgamma (eEF1Bγ) [29] and two temporary resident proteins, Pex19p shuttle protein [30] and the Vps23p adaptor ESCRT protein [28], [31], [32]. Although the functions of several of these proteins have been studied in some details, additional host proteins might be also present in tombusvirus VRC [5], [10], [21]–[23], [25]. One of the intriguing questions is if tombusviruses use host-coded helicases for their replication. This is because, unlike larger RNA viruses, the tombusvirus genome does not code for a protein with helicase function. Thus, can tombusviruses and other small RNA viruses replicate without the use of a helicase or they subvert a cellular helicase(s) to assist their replication?

DEAD-box proteins constitute the largest family of RNA helicases that are involved in all aspects of cellular metabolism and perform RNA duplex unwinding and remodeling of RNA-protein complexes in cells [33]–[35]. Ded1p, which is essential for yeast growth, is among the best-characterized DEAD-box helicases [36]. It is involved in initiation of translation of every yeast mRNA [37]–[39] and down-regulation of Ded1p level also reduced p33 and p92 levels in yeast [20]. Therefore, not surprisingly, TBSV replication also decreased significantly in yeast with reduced level of Ded1p. Due to its essential role in translation, it is critical to separate the possible role of Ded1p in viral replication from its effect on viral translation.

In this paper, a yeast-based cell-free TBSV replication assay and recombinant Ded1p was used to test the role of Ded1p in TBSV replication. We define that Ded1p plays a role in enhancing plus-strand (+)RNA synthesis by the viral replicase. We also find that Ded1p is a component of the tombusvirus VRC and binds to the 3′-end of the viral minus-stranded (−)RNA. The obtained data support the model that Ded1p unwinds the 3′-end of the TBSV (−)RNA, rendering the RNA compatible for initiation of (+)-strand synthesis. Interestingly, we find that Ded1p and GAPDH play non-overlapping functions to enhance (+)-strand synthesis. Altogether, the two host factors enhance TBSV replication synergistically by interacting with the viral (−)RNA and the replication proteins.

## Results

### Reduced level of TBSV RNA replication in yeast cell-free extract depleted for Ded1p DEAD-box RNA helicase

Since Ded1p is an essential translation factor for all yeast mRNAs [38], [39], it is difficult to separate its direct versus indirect effect on TBSV replication in yeast model host. To circumvent this problem, we used whole cell extracts (CFE) prepared from yeast containing reduced level of Ded1p (Figure S1) to support cell-free TBSV replication. As we have shown previously, TBSV (+)RNA can perform one complete cycle of replication in the CFE-based replication assay when purified recombinant p33 and p92^pol^ replication proteins are provided [23], [40]. The CFE-based replication assay showed that both (+) and (−)RNA synthesis decreased by over 4-fold when Ded1p was down-regulated as compared with the control CFE prepared from yeast with high level of Ded1p expression (Figure S1, lanes 4–6 versus 1–3). Thus, these data confirm that Ded1p is important for TBSV replication. However, the observed decrease in the CFE-based TBSV replication could be due to either reduced replication when Ded1p is limiting (direct effect) or lesser amounts of other critical host factors needed for TBSV replication in the CFE with reduced level of Ded1p (indirect effect due to Ded1p's role in host translation). To address these points, we first studied if Ded1p is present within the tombusvirus replicase and then, what is the mechanistic role of Ded1p during TBSV RNA synthesis.

### Ded1p is co-purified with the tombusvirus replicase

**Figure 1:**
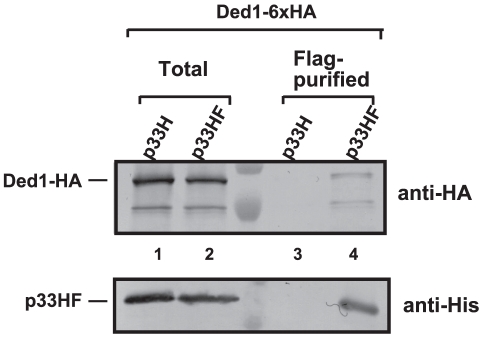
Co-purification of Ded1p with the p33 replication protein from yeast. The FLAG/6×His-tagged p33HF or 6×His-tagged p33H was purified from yeast extracts using a FLAG-affinity column (lanes 3–4). Top panel: Western blot analysis of Ded1p tagged with 6×HA, expressed from the natural promoter in the chromosome, with anti-HA antibody in the purified p33 preparations. Bottom panel: Western blot analysis of HF-tagged p33 or 6×His-tagged p33 with anti-His antibody. Note that “total” represents the total protein extract from yeast expressing the shown proteins. Each experiment was repeated three times.

To examine if Ded1p is present within the tombusvirus replicase complex, we FLAG affinity-purified the tombusvirus replicase from yeast cells actively replicating TBSV repRNA [14], [28]. This yeast also expressed HA-tagged Ded1p from yeast chromosome based on the native promoter. We found that the tombusvirus replicase preparation, which is highly active on added templates *in vitro* (not shown), contained Ded1p (Figure 1, lane 4), while Ded1p was undetectable in the control yeast sample obtained using the same affinity purification (Figure 1, lane 3). The control yeast also expressed the tombusvirus replication proteins (including the 6×His-tagged p33, but lacking FLAG-tag) and the HA-tagged Ded1p from yeast chromosome based on the native promoter and supported TBSV repRNA replication (not shown).

We also FLAG-affinity-purified 6×His/FLAG-tagged Ded1p from the membrane-fraction of yeast co-expressing HA-p33 and HA-p92 and found that the purified Ded1p preparation contained HA-tagged p33 (Figure S2A, lane 1). The affinity-purified Ded1p preparation also showed TBSV replicase activity *in vitro* on added TBSV template (Figure S2B, lane 2), suggesting that the membrane-bound Ded1p is associated with the active tombusvirus replicase. Altogether, these data support the model that Ded1p is part of the tombusvirus replicase.

### Recombinant Ded1p DEAD-box helicase enhances TBSV RNA replication in yeast cell-free extract

**Figure 2:**
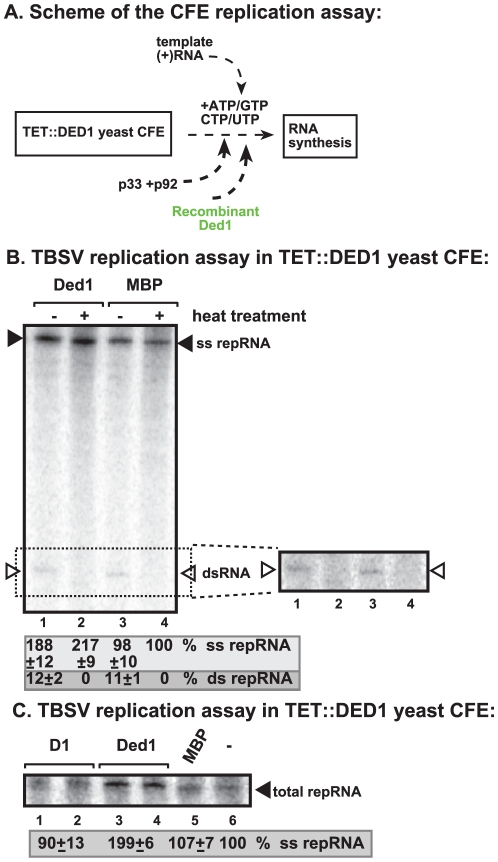
Cell-free TBSV replication assay supports a role for Ded1p helicase in plus-strand synthesis. (A) Scheme of the CFE-based TBSV replication assay. Purified recombinant p33 and p92^pol^ replication proteins of TBSV and *in vitro* transcribed TBSV DI-72 (+)repRNA were added to the whole cell extract prepared from Ded1p-depleted yeast strain. The assay also contained purified recombinant Ded1p (0.3 µg) or MBP (the same molar amount as Ded1p) as a control. (B) Left panel: Detection of single- and double-stranded RNA products produced in the cell-free TBSV replication assay. The dsRNA products are also shown on the right subpanel with high contrast for better visualization. Odd numbered lanes represent replicase products, which were not heat treated (thus both ssRNA and dsRNA products are present), while the even numbered lanes show the heat-treated replicase products (only ssRNA is present). The % of dsRNA and ssRNA in the samples are shown. Note that, in the nondenatured samples, the dsRNA product represents the annealed (−)RNA and the (+)RNA, while the ssRNA products represents the newly made (+)RNA products. Right panel: A higher contrast image of portion of the left panel to aid the visualization of the dsRNA products. Each experiment was repeated three times. (C) Denaturing PAGE analysis of the ^32^P-labeled TBSV repRNA products obtained in the CFE assay in the presence of ATPase inactive D1 mutant of Ded1p (0.3 µg), wt Ded1p (0.3 µg) or MBP. Each experiment was repeated three times.

To address if Ded1p could play a direct role in TBSV replication, we added purified recombinant Ded1p to yeast CFE with reduced level of Ded1p (Figure 2A). We observed a ∼2-fold increased level of TBSV RNA replication (based on total single-stranded repRNA level) in the presence of recombinant Ded1p when compared with the MBP control (Figure 2B, lane 2 versus 4). Interestingly, the amount of double-stranded RNA, which correlates with (−)-strand levels [23], did not increase in the presence of Ded1p in the CFE assay (Figure 2B, lanes 1 versus 3). This finding suggests that (−)-strand RNA did not contribute to the 2-fold increase in total TBSV RNA levels when recombinant Ded1p was added to the CFE-based replication assay. Therefore, we conclude that the added recombinant Ded1p selectively increased TBSV (+)-strand RNA synthesis *in vitro*.

To demonstrate if the ATPase/helicase activity of Ded1p is important for the enhancement of TBSV RNA replication in the CFE-based replication assay, we tested an ATPase defective mutant of Ded1p (D1 mutant) [41]. This mutant could not enhance TBSV replication *in vitro* (Figure 2C, lanes 1–2 versus 3–4), suggesting that the ATPase activity of Ded1p is important for TBSV replication.

### Ded1p selectively promotes TBSV (+)RNA synthesis in yeast cell-free extract

**Figure 3 ppat-1002537-g003:**
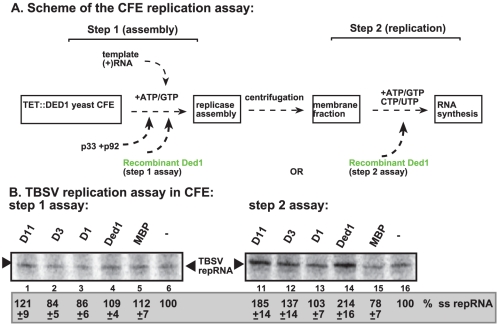
Step-wise cell-free TBSV replication assay does not support a role for Ded1p helicase in the assembly of the TBSV VRC. (A) Scheme of the CFE-based TBSV replicase assembly and replication assays. Purified recombinant p33 and p92^pol^ replication proteins of TBSV and *in vitro* transcribed TBSV DI-72 (+)repRNA were added to the whole cell extract prepared from Ded1p-depleted yeast strain in step 1. The assay either contained or lacked the purified recombinant Ded1p (0.3 µg) or MBP during step 1. Note that the assay was performed in the presence of ATP/GTP to facilitate TBSV VRC assembly, but prevent RNA synthesis in step 1. After step 1, centrifugation was used to collect the membrane fraction of the CFE, and after washing the membranes, step 2 was performed in the presence of ATP/CTP/GTP and ^32^P-UTP to allow TBSV RNA replication. In the samples presented in right panel, Ded1p or MBP were added at the beginning of step 2. (B) Denaturing PAGE analysis of the ^32^P-labeled TBSV repRNA products obtained in the CFE assays in the presence of wt and various Ded1p mutants (0.3 µg each) (Figure S3C) or MBP. See further details in Figure 2. Note that the various preps of D3 mutant of Ded1p showed high variation in *in vitro* activity (for reasons currently unknown)- see Figure 4B. Each experiment was repeated at least three times and the data were used to calculate standard deviation.

To further test what step(s) Ded1p promotes during TBSV replication, we performed a two-step replication assay based on yeast CFE. In this assay, the first step includes the assembly of the replicase complex on the endogenous membranes present in CFE in the presence of the viral (+)repRNA, the p33/p92 replication proteins and ATP/GTP [23] (Figure 3A, step 1). Under these conditions, the viral replication proteins recruit the repRNA to the membrane and the replicase becomes partially RNase and protease insensitive, but it cannot initiate minus-strand synthesis yet, due to the absence of CTP/UTP [23]. Then, centrifugation and washing the membranes will remove all the proteins and molecules not bound to the membrane. This is followed by addition of ATP/CTP/GTP/UTP to initiate RNA synthesis during the second step.

Addition of purified Ded1p to the CFE during the first step did not increase TBSV replication (Figure 3B, lane 4 versus 5). This suggests that Ded1p did not facilitate the assembly of the replicase complex, unlike other host factors, such as Hsp70 and eEF1A [23], [27]. Moreover, Ded1p was likely lost during the centrifugation/washing step in this assay due to its low association with the membrane. However, addition of Ded1p exclusively during the second step of the CFE assay resulted in ∼2-fold increase in TBSV RNA replication (Figure 3B, lane 14 versus 15), similar to the stimulatory effect of Ded1p during standard CFE replication assay (Figure 2). Interestingly, a Ded1p mutant (D1) deficient in ATPase activity could not stimulate TBSV RNA synthesis in this assay (Figure 3B, lane 13), while a mutant (D11, Figure S3C) with increased ATPase activity [41] promoted TBSV RNA synthesis by ∼2-fold (Figure 3B, lane 11). Altogether, these findings indicate that Ded1p has a direct stimulatory function during TBSV RNA synthesis, while Ded1p is unlikely to affect viral RNA recruitment for replication or VRC assembly.

### Ded1p enhances TBSV (+)RNA synthesis by the purified tombusvirus replicase

**Figure 4:**
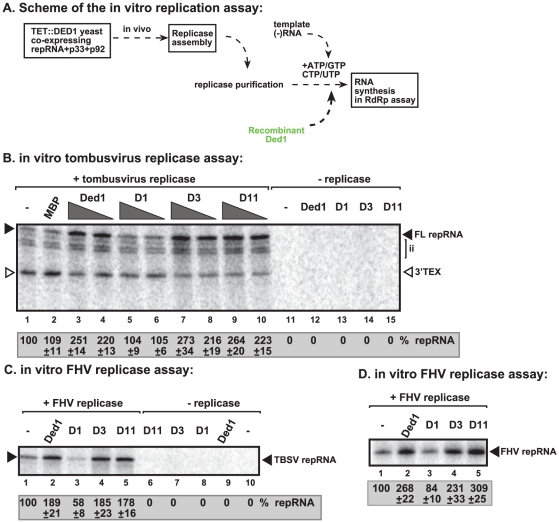
Ded1p promotes plus-strand synthesis by the affinity-purified tombusvirus and FHV replicases. (A) Scheme of the tombusvirus replicase assay. Yeast with depleted Ded1p co-expressing p33 and p92^pol^ replication proteins and DI-72 (+)repRNA were used to affinity-purify the RNA-free tombusvirus replicase. The *in vitro* assays were programmed with DI-72 (−)repRNA, and they also contained purified recombinant Ded1p, mutants or MBP in addition to ATP/CTP/GTP and ^32^P-UTP. (B) Representative denaturing gel of ^32^P-labeled RNA products synthesized by the purified tombusvirus replicase *in vitro* in the presence of 0.5 µg or 1.0 µg of purified recombinant Ded1p or its mutants is shown. The level of complementary RNA synthesis producing “repRNA” (marked as “FL”, the full-length product, made via *de novo* initiation from the 3′-terminal promoter) in each sample was compared to that of the replicase activity obtained in the absence of added recombinant protein (lane 1). Note that this replicase preparation also synthesizes *de novo* internal initiation products (“ii”) and 3′-terminal extension products (“3′TEX”). Each experiment was repeated three times. (C) Representative denaturing gel of ^32^P-labeled RNA products synthesized by the purified FHV replicase *in vitro* in the presence of 1.0 µg of purified recombinant Ded1p or its mutants is shown. Note that the FHV replicase can use TBSV (−)repRNA as a template *in vitro*. Each experiment was repeated three times. (D) Representative denaturing gel of ^32^P-labeled RNA products synthesized by the purified FHV replicase *in vitro* in the presence of 1.0 µg of purified recombinant Ded1p or its mutants is shown. Note that the FHV DI-634 (−)repRNA was used as a template in the FHV replicase assay.

To further test the direct effect of Ded1p on RNA synthesis, we utilized detergent-solubilized and affinity-purified tombusvirus replicase from Ded1p-depleted yeast (Figure 4A). This purified replicase can only synthesize complementary RNA products on added TBSV templates, but, unlike the above membrane-bound replicase in the CFE-based assay, it cannot perform a complete cycle of RNA synthesis [14], [15].

We found that addition of purified recombinant Ded1p to the purified tombusvirus replicase programmed with the (−)repRNA stimulated (+)-strand synthesis by ∼2.5–3-fold (Figure 4B, lanes 3–4 versus 1–2; and Figure S3D, lane 3 versus 2). Interestingly, Ded1p stimulated the production of the full-length (+)-strand RNA product, while the amount of 3′-terminal extension product (3′TEX; due to self-priming by the 3′ end of the template [42]–[44], Figure S3B) decreased in the presence of added recombinant Ded1p. Therefore, we suggest that Ded1p facilitates the *de novo* initiation on the (−)RNA template by the tombusvirus replicase.

The ATPase inactive mutant D1 (Figure 4B, lanes 5–6 and S3D, lane 4), D5 and D10 (Figure S3D, lanes 6 and 8) could not promote (+)-strand synthesis, suggesting that the ATPase activity of Ded1p is required for the above stimulatory effect on TBSV RNA synthesis. Also, two active ATPase mutants, D3 and D11 (Figure S3C), did facilitate (+)-strand synthesis (Figure S3D, lanes 5 and 9), confirming that the ATPase activity of Ded1p is required during TBSV (+)-strand RNA synthesis. Interestingly, Ded1p mutant D11, albeit has increased ATPase activity, has wt-like strand displacement activity *in vitro* [41]. We found that D11 behaved similarly in TBSV replicase assay (Figure S3D) to wt Ded1p, suggesting that the unwinding activity of Ded1p is important during TBSV replication.

To test if Ded1p can also stimulate the activity of the tombusvirus replicase on (+)-stranded RNA templates, we used DI-72(+) RNA in the purified tombusvirus replicase assay (Figure S4A). The TBSV (+)RNA is known to carry a replication silencer element (RSE) at the 3′ end that inhibits (−)RNA synthesis *in vitro* [45]. Addition of Ded1p to the tombusvirus replicase assay did not enhance (−)RNA synthesis (Figure S4B, lane 3 versus 1–2). Also, D1 mutant deficient in ATPase activity had not much effect on (−)RNA synthesis (Figure S4B, lane 4 versus 1–2). Based on these data, we suggest that Ded1p does not affect (−)RNA synthesis by the tombusvirus replicase *in vitro*.

### Ded1p binds to the 3′ end of the TBSV (−)RNA *in vitro*

**Figure 5:**
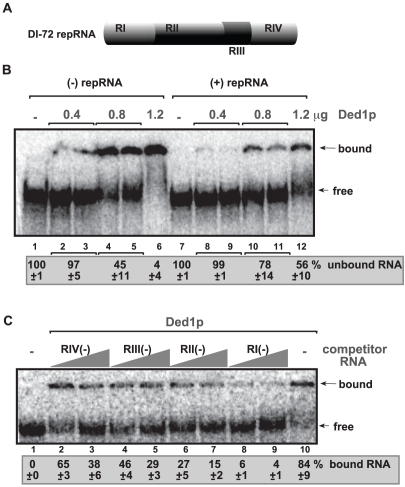
Ded1p binds to the 3′ end of the TBSV (−)RNA. (A) Schematic representation of the four regions in DI-72 repRNA used in the binding assay. (B) *In vitro* binding assay with purified Ded1p using ^32^P-labeled ssRNA templates. The assay contained the (−) or (+) DI-72 repRNA (∼0.1 pmol) plus increasing amount [0.4 µg (lanes 2–3 and 8–9), 0.8 µg (lanes 4–5 and 10–11) and 1.2 µg (lanes 6 and 12)] of purified recombinant Ded1p. The free or Ded1p-bound ssRNA was separated on nondenaturing 5% acrylamide gels. (C) RNA gel shift analysis shows that Ded1p binds the most efficiently to RI(−). ^32^P-labeled DI-72 (−)repRNA template (∼0.1 pmol) and unlabeled competitor RNAs (3 and 6 pmol) representing one of the four regions (see panel A) were used in the competition assay. The Ded1p - ^32^P-labeled ssRNA complex was visualized on nondenaturing 5% acrylamide gels. Each experiment was repeated at least three times.

To identify the region(s) of the TBSV RNA bound by the recombinant Ded1p, we performed electrophoresis mobility shift assay (EMSA) with purified components. Comparison of ^32^P-labeled TBSV (+) and (−)RNAs in binding to purified recombinant Ded1p revealed that (−)RNA bound more readily to Ded1p *in vitro* than (+)RNA (Figure 5B, lanes 2–6 versus 8–12). Additional EMSA experiments using the four regions in DI-72 (−)repRNA (Figure 5A) as unlabeled competitors revealed that RI(−) was the most efficient in outcompeting the ^32^P-labeled TBSV (−)repRNA in binding to Ded1p (Figure 5C, lanes 8–9). This is important since RI(−) is the 3′ end of (−)repRNA and contains important cis-acting elements, such as the promoter and a short enhancer sequence for (+)RNA synthesis [46], [47].

Further testing of Ded1p binding to (+)repRNA regions revealed that RIV(+), representing the 3′ noncoding region in TBSV RNA, was bound more efficiently by Ded1p (Figure S5, lane 11) than the other regions of DI-72(+) RNA were (lanes 2–9). However, the binding of Ded1p to RIV(+) was readily outcompeted by the unlabeled DI-72 (+)repRNA (Figure S5, lane 12).

To test if Ded1p can interact with the TBSV p33 replication protein, we used the membrane-based split-ubiquitin assay [31]. We found that Ded1p interacted with p33 protein in yeast (Figure S6A). The pull-down experiments with MBP-p33 and MBP-p92 also showed that Ded1p bound to the tombusvirus replication proteins *in vitro* (Figure S6B). This was further supported by the reverse pull-down experiments with GST-tagged Ded1p, which resulted in the co-purification of p33 and p92 (Figure S6C).

### RH20, a DEAD-box helicase from Arabidopsis stimulates TBSV (+)RNA synthesis by the purified tombusvirus replicase

**Figure 6:**
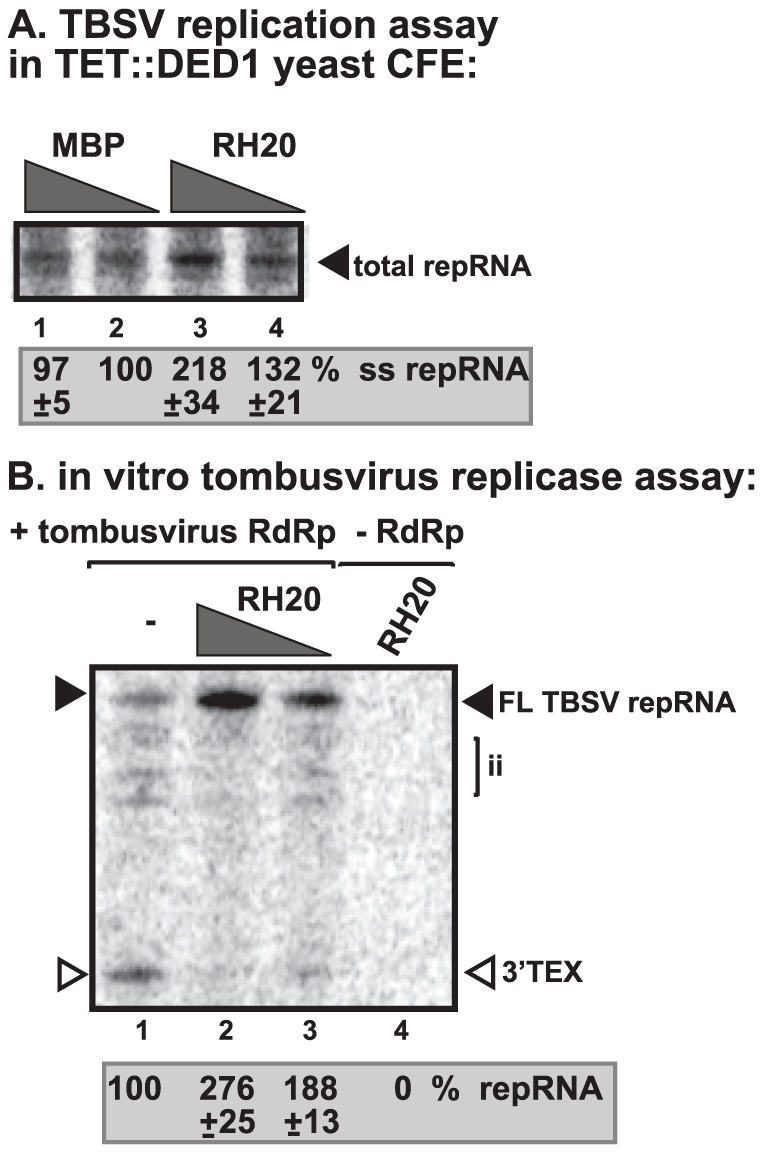
AtRH20 DEAD-box helicase promotes TBSV repRNA replication in the CFE-based assay. (A) Representative denaturing gel of ^32^P-labeled TBSV repRNA products obtained in the CFE assay prepared from Ded1p-depleted yeast strain in the presence of 0.2 µg (lane 3) or 0.3 µg (lane 4) of purified recombinant AtRH20 is shown. The experiment was repeated three times. (B) AtRH20 DEAD-box helicase promotes plus-strand synthesis by the affinity-purified tombusvirus replicase. Representative denaturing gel of ^32^P-labeled RNA products synthesized by the purified tombusvirus replicase *in vitro* in the presence of 0.5 µg (lane 3) or 1.0 µg (lanes 2, 4) of purified recombinant AtRH20 is shown. Yeast with depleted Ded1p co-expressing p33 and p92^pol^ replication proteins and DI-72 (+)repRNA were used to affinity-purify the RNA-free tombusvirus replicase. The *in vitro* assays were programmed with DI-72 (−)repRNA, and they also contained purified recombinant AtRH20 (Lanes 2–4) in addition to ATP/CTP/GTP and ^32^P-UTP. Lane 4 represents the sample with no tombusvirus replicase added. The experiment was repeated three times.

To test if a DEAD-box helicase from plants might have similar stimulatory function on the activity of tombusvirus replicase, we have cloned and purified RH20 cytosolic DEAD-box helicase from *Arabidosis thaliana*, which shows high degree of similarity to yeast Ded1p (Figure S7). Addition of the recombinant AtRH20 to the CFE prepared from Ded1p-depleted yeast strain increased TBSV repRNA replication by ∼2-fold (Figure 6A, lane 3). Also, adding the recombinant RH20 to the replicase assay led to almost 3-fold increase of the activity of the purified tombusvirus replicase on TBSV (−)RNA template (Figure 6B, lanes 2–3 versus 1). To test if AtRH20 can interact with the TBSV p33 replication protein, we used the membrane-based split-ubiquitin assay [31]. We found that AtRH20, similar to Ded1p, interacted with p33 protein in yeast (Figure S6A). Altogether, these data strongly suggest that plants also have DEAD-box helicases that could play similar role to the yeast Ded1p DEAD-box helicase in tombusvirus (+)RNA synthesis.

### Ded1p enhances Flock house virus (+)RNA synthesis by the purified replicase

To test if Flock house virus (FHV) replicase is affected by Ded1p, first we have developed an *in vitro* FHV replication assay based on affinity-purified FHV replicase preparation that can be programmed with exogenously added RNAs (Figure 4C, lanes 1 versus 10). The *in vitro* FHV replicase assay revealed that the purified recombinant Ded1p increased (+)-strand RNA synthesis on the (−)-stranded FHV template by ∼3-fold (Figure 4D, lanes 2 versus 1) and by ∼2-fold on the heterologous TBSV (−)repRNA template (Figure 4C, lane 2 versus 1). Addition of D1 ATPase inactive mutant of Ded1p decreased RNA synthesis by the FHV replicase by 20–40% (Figure 4C, lane 3; Figure 4D, lane 3), confirming that the ATPase activity of Ded1p is important for the stimulatory function on (+)RNA synthesis. Ded1p did not stimulate (−)RNA synthesis by the FHV replicase on (+)-stranded TBSV RNA (Figure S4C, lane 3 versus 1–2) or FHV (+)RNA (Figure S4D, lane 2 versus 1). It is likely that the observed stimulation of FHV replicase activity by Ded1p is direct, since we found that Ded1p bound to the FHV repRNA (Figure S8A) and protein A RdRp protein *in vitro* (Figure S8B). Altogether, these data show that, similar to the tombusvirus replicase, the activity of FHV replicase is stimulated by Ded1p only on the (−)RNA templates, but not when using the (+)RNA templates.

### Ded1p facilitates initiation by the tombusvirus replicase on RNA/DNA duplex

**Figure 7:**
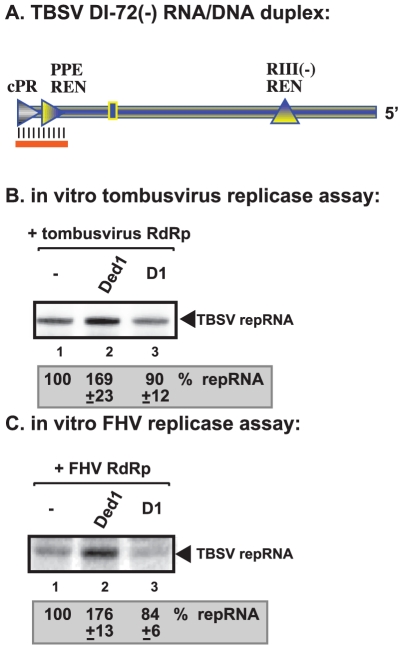
Ded1p facilitates the RNA synthesis by the tombusvirus and FHV replicases on short partial DNA/RNA duplex. (A) Schematic representation of the DNA/RNA duplex used in the replicase assays. The template consists of DI-72 (−)repRNA, whose 3′ end forms a 21 nt duplex with a complementary DNA oligo as shown. The following cis-acting sequences involved in (+)-strand synthesis are shown: cPR is an 11 nt long sequence used as a (+)-strand initiation promoter; PPE REN is a promoter proximal replication enhancer; while RIII(−)REN is a strong replication enhancer within RIII(−) sequence. (B) Representative denaturing gel of ^32^P-labeled RNA products synthesized by the purified tombusvirus replicase *in vitro* in the presence of 1.0 µg of purified recombinant Ded1p or D1 mutant. The level of complementary RNA synthesis using the DNA/RNA duplex (panel A) in each sample was compared to that of the replicase activity obtained in the absence of added recombinant protein (lane 1). See further details in Figure 4. (C) Representative denaturing gel of ^32^P-labeled RNA products synthesized by the purified FHV replicase *in vitro* in the presence of 1.0 µg of purified recombinant Ded1p or D1 mutant on the DNA/RNA duplex (panel A). Each experiment was repeated three times.

To gain further insights into the mechanism of Ded1p-driven stimulation of TBSV and FHV RNA synthesis, we exploited template structures, such as an RNA/DNA duplex, that are known to hinder RdRp-driven RNA synthesis [48], [49]. Since Ded1p is an RNA helicase [39], [41] and the ATPase/helicase function of Ded1p is needed for stimulation of (+)RNA synthesis by the tombusvirus and FHV replicases, we wanted to examine if Ded1p might facilitate RNA synthesis on a partial DNA/RNA duplex. We chose partial duplex for this assay, since Ded1p and other DEAD-box helicases are not processive enzymes and can only unwind short duplexes [34]. Also, we have shown previously that short DNA oligos hybridized to the promoter region of (−)RNA can inhibit (+)RNA synthesis by the tombusvirus replicase *in vitro* [42], [47], [48]. Interestingly, addition of purified Ded1p to the tombusvirus replicase assay containing the short RNA/DNA duplex (Figure 7A) enhanced (+)RNA synthesis by ∼70% (Figure 7B, lane 2 versus 1). Similarly, Ded1p also promoted (+)RNA synthesis by the FHV replicase using the same RNA/DNA duplex (Figure 7C, lane 2 versus 1). D1 ATPase deficient mutant could not stimulate RNA synthesis on the RNA/DNA duplex by tombusvirus or FHV replicases (Figure 7B–C, lane 3). Thus, the ATPase/helicase function of Ded1p is needed for the stimulatory effect on RNA synthesis on a short RNA/DNA duplex, suggesting that RNA unwinding by the Ded1p is a function provided by this host factor.

### Non-overlapping functions of Ded1p and GAPDH in promoting initiation by the tombusvirus replicase

**Figure 8:**
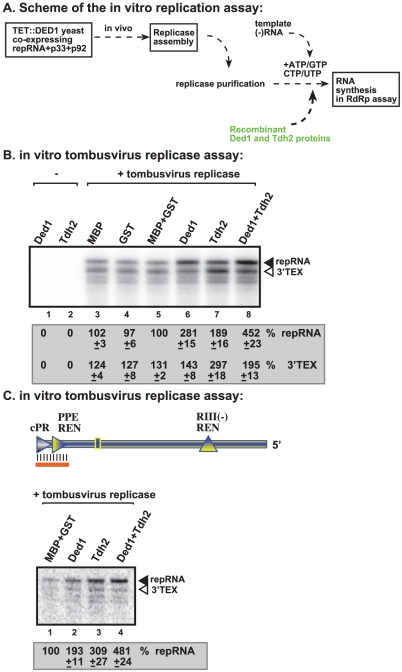
Ded1p and GAPDH (Tdh2p) act synergistically to enhance RNA synthesis by the tombusvirus replicase. (A) Schematic representation of the replicase assay used. The purified recombinant proteins were added at the same time to the assay. (B) Representative denaturing gel of ^32^P-labeled RNA products synthesized by the purified tombusvirus replicase *in vitro* in the presence of 1.0 µg of purified recombinant MBP-Ded1p and/or 0.4 µg of GST-Tdh2p on DI-72 (−)repRNA template. The level of complementary RNA synthesis in each sample was compared to that of the replicase activity obtained in the presence of GST and MBP recombinant proteins (lane 5) (used in comparable molar amounts to MBP-Ded1p and GST-Tdh2p). The ^32^P-labeled RNA products were RNase One treated to visualize the 3′terminal extension products (“3′TEX”) better. Note that the RNAse One treatment causes a shift in migration of the 3′TEX product (compare with Figure 4B, untreated samples). See further details in Figure 4. (C) Representative denaturing gel of ^32^P-labeled RNA products synthesized by the purified tombusvirus replicase *in vitro* in the presence of 1.0 µg of purified recombinant MBP-Ded1p and/or 0.4 µg of GST-Tdh2p on the DNA/RNA duplex (Figure 7 panel A). Each experiment was repeated three times.

GAPDH (Tdh2p in yeast) RNA binding protein is also a host factor stimulating (+)RNA synthesis by the tombusvirus replicase [25], [50]. To test if Ded1p and GAPDH could play a complementary role during (+)RNA synthesis, we added the purified recombinant Ded1p and Tdh2p to the *in vitro* tombusvirus replicase assay based on the purified preparation (Figure 8A). While Ded1p mostly stimulated *de novo* (+)RNA synthesis initiated from the 3′ end of the (−)repRNA up to ∼3-fold (Figure 8B, lane 6), Tdh2p enhanced both *de novo* (+)RNA synthesis and 3′TEX by ∼2- and ∼3-fold, respectively (Figure 8B, lane 7). Interestingly, the two host proteins together had the largest (4.5-fold) effect on (+)RNA synthesis, while their effect on 3′TEX was only ∼2-fold. Based on these data, we suggest that Ded1p and Tdh2p have a synergistic effect on (+)RNA synthesis by promoting *de novo* initiation from the 3′ end of the (−)RNA template.

We also performed similar experiments with a short partial RNA/DNA duplex as a template. Both Ded1p and Tdh2p alone could enhance (+)RNA synthesis on the short RNA/DNA duplex (Figure 8C, lanes 2–3 versus 1) but the largest stimulatory effect (i.e., close to 5-fold increase) was seen when both host factors were included in the assay (Figure 8C, lane 4). Altogether, these data further support that Ded1p and GAPDH play synergistic roles in (+)RNA synthesis by the tombusvirus replicase.

## Discussion

RNA viruses form several different ribonucleic acid-protein complexes (RNP) in infected cells during various steps of infection [9]. Since the viral RNA plays multiple roles during infection, it is likely that remodeling of the viral RNAs and RNP complexes during the switch from one step to another requires RNA helicases or RNA chaperones. Accordingly, the larger RNA viruses all code for RNA helicases [51], [52]. However, RNA viruses with shorter than 6 kB genomes usually do not code for RNA helicases. They could still use RNA helicases during infections if they can subvert selected host helicases for viral purposes. Indeed, we show here that Ded1p helicase is recruited for TBSV replication to aid (+)-strand RNA synthesis.

It is not yet known if Ded1p is the only host helicase needed for TBSV replication, since genome-wide screens and global proteomics approaches with TBSV have identified additional host helicases as well [9], [10], [28]. However, Ded1p helicase seems to be an ideal host factor to be recruited for viral replication because it is involved in mRNA and viral RNA translation, thus it is co-localized with the viral RNA prior to replication. Also, by recruiting Ded1p, viruses could affect RNA degradation (P-body formation; [53]) and initiation of translation of new RNAs, likely affecting subsequent host translation (including virus-induced mRNAs coding for anti-viral proteins or required for anti-viral signaling).

The essential nature of Ded1p for host mRNA translation, however, makes characterization of Ded1p function as a host factor difficult. Therefore, the combined use of *in vivo* and *in vitro* approaches might be necessary to dissect the function of Ded1p in RNA virus replication as shown in this paper. By using a Ded1p-depleted yeast CFE in combination with recombinant Ded1p allowed us to define that the ATPase (helicase) activity of Ded1p is required for efficient TBSV (+)-strand synthesis (see below). Ded1p also increased the RdRp activity of the FHV replicase, suggesting that recruitment of DEAD-box helicases might also be useful for additional small RNA viruses (see below).

### Ded1p helicase is a component of the tombusvirus replicase complex

Co-purification experiments revealed that Ded1p is present in the tombusviral VRC (Figure 1). In addition, Ded1p affected (+)-strand synthesis, but not the assembly of the VRC *in vitro* (Figures 2–4), indicating that Ded1p is likely present in the VRC. Surprisingly, Ded1p, unlike other previously tested host factors Hsp70, GAPDH, or eEF1A [21]–[23], [27], [54], was able to affect TBSV repRNA replication even after the replicase assembly step took place *in vitro* (Figure 3). This suggests that Ded1p can enter the membrane-bound VRC, possibly due to its interaction with p33 (Figure S6) and the helicase activity of Ded1p might lead to some remodeling of VRC.

It is not yet known if additional members of the large helicase family could perform similar function to Ded1p during TBSV replication. Our *in vitro* data with AtRH20 plant helicase protein, which is very similar to Ded1p (Figure S7), suggests that this protein can likely perform similar function in plant infections. For example, AtRH20 has been shown to boost TBSV (+)RNA synthesis *in vitro* (Figure 6) and bind to p33 replication protein (Figure S6A), which can facilitate its recruitment into the VRC. Since more than 50 RNA helicases are present in plants, further experiments will be needed to define if additional host helicases might also be involved in TBSV replication.

### Role of Ded1p helicase in stimulation of TBSV RNA synthesis

The *in vitro* data, based on the CFE assay containing the membrane-bound VRC as well as the solubilized/purified tombusvirus replicase, showed the Ded1p can mainly stimulate TBSV (+)-strand synthesis, while its effect on (−)RNA synthesis is less pronounced. The ATPase activity of Ded1p is required for this stimulatory effect, suggesting that the helicase function of Ded1p is likely important for unwinding the secondary structure of (−)RNA template with the purified tombusvirus replicase or destabilizing the replication intermediate with the membrane-bound VRC, which might contain dsRNA structure. This function of Ded1p can explain why yeast with down-regulated Ded1p level produced small amount of (+)-stranded RNA progeny, albeit it has also shown that depleted Ded1p reduced the level of p33/p92 in yeast [20]. However, the decreased p33/p92 levels are expected to reduce both (+) and (−)RNA levels [55] (Figure S1). Since the recombinant Ded1p enhanced (+)-strand synthesis by the purified recombinant tombusvirus replicase, we propose that Ded1p directly affect TBSV RNA synthesis via affecting the structure of the RNA templates. However, we cannot exclude that Ded1p could also affect the activity of the VRC due to its interaction with p33, albeit the assembly of the VRC was not affected by Ded1p in the CFE-based assay (Figure 3). Overall, the recruitment of a host DEAD-box helicase for replication of a small RNA virus is remarkable, since RNA viruses with less than 6 kB genomes are usually do not code for their own helicases [51]. These viruses are thought to replicate without needing a helicase by using RNA chaperones or possibly recruiting host helicases. The emerging picture with TBSV is that this virus utilizes both the viral-coded p33 RNA chaperone [48] and the host Ded1p helicase for replication by promoting (+)-strand synthesis. Both p33 and Ded1p have been shown to open up short DNA/RNA duplexes, although the activity of Ded1p was more robust than that of p33 *in vitro* [48]. Why would TBSV utilize both an RNA chaperone and an RNA helicase? It is possible that both proteins are needed for robust (+)RNA synthesis to make excess amount of progeny RNA. Also, Ded1p helicase could be involved in remodeling the viral RNA bound by the viral RdRp or host proteins prior or during RNA synthesis. Since Ded1p can work in both 5′-to-3′ and 3′-to-5′ directions [56], it could be used for multiple purposes during RNA synthesis.

### A model on the synergistic roles of host factors in TBSV (+)RNA synthesis

**Figure 9:**
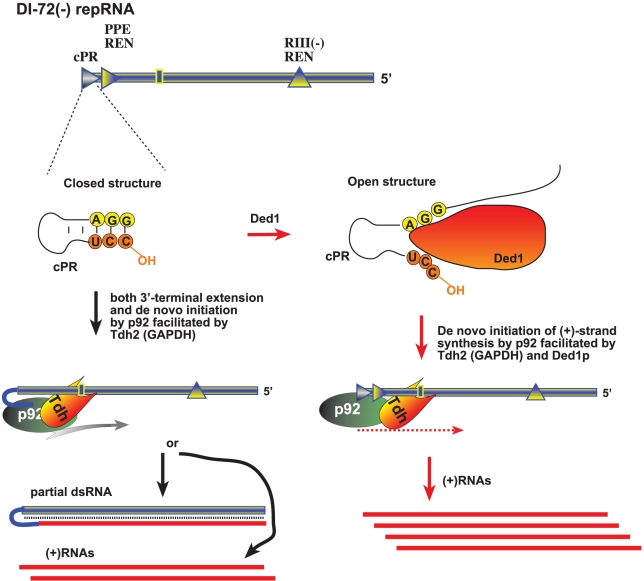
A model describing the functions of Ded1p and GAPDH during tombusvirus replication. The (−)repRNA, which is shown with a “closed” 3′ end due to either a secondary structure within the promoter (cPR) in the (−)repRNA or part of a dsRNA structure, could be unwound by Ded1p around the 3′ end, making the (−)repRNA “open”. The open ssRNA structure would facilitate (+)RNA synthesis by the tombusvirus p92 RdRp protein. GAPDH could facilitate (+)RNA synthesis via binding to a 3′ AU-rich sequence (indicated as a box) and also bringing in the p92 protein and positioning it over the cPR promoter as shown. Thus, Ded1p and GAPDH might play a complementary role, leading to synergistic effect on (+)RNA synthesis. In the absence of Ded1p, GAPDH could promote both 3′-terminal extension and (+)RNA synthesis by facilitating the positioning of p92 over the 3′ end.

Based on the available data, it seems that TBSV (+)RNA synthesis is not only affected by the p33/p92 replication proteins, but by GAPDH and Ded1p host proteins as well. Since the viral replication proteins were shown to bind to TBSV (−)RNA nonspecifically [57], we propose that the above host proteins, which bind strongly to the TBSV (−)repRNA, are involved in facilitating the proper and efficient recruitment of the p92 RdRp protein to the (−)RNA template (or alternatively to the dsRNA intermediate) within the VRC as shown in Figure 9. For example, Ded1p might unwind either local secondary structure in (−)repRNA or dsRNA region and that, in turn, could favor binding of GAPDH or p92 to the (−)RNA template. This is followed by binding of GAPDH to an AU-rich internal site and proper positioning of the p92 RdRp (bound to GAPDH, [50]) over the (+)-strand initiation promoter, leading to (+)RNA synthesis. The synergistic effect of these host proteins could promote efficient recycling of the viral RdRp resulting in multiple rounds of (+)RNA synthesis (Figure 9). Thus, the roles of these host proteins are to serve as “matchmakers” between the viral RNA template and the viral RdRp. It is intriguing that two host proteins, eEF1A and eEF1Bγ, are proposed to serve somewhat similar functions during (−)RNA initiation for TBSV [27], [29]. Therefore, the emerging picture is that TBSV utilizes different host proteins for promoting (−) versus (+)RNA synthesis. This strategy could be beneficial for the virus by allowing asymmetric RNA synthesis, thus leading to excess amount of progeny (+)RNA.

Overall, host DEAD-box or related RNA helicases have been shown to affect many different aspects of virus infections, including translation of viral proteins [58]–[60]; viral RNA replication [61]–[64]; reverse transcription [65]; the activity of anti-viral proteins [66], [67], and virus-mediated regulation of host gene transcription [68]. Interestingly, Ded1p is known to affect minus-strand synthesis during replication of the L-A dsRNA virus of yeast [69], suggesting that the use of DEAD-box helicases is wide-spread among RNA viruses.

## Materials and Methods

### Yeast strains and expression plasmids

*Saccharomyces cerevisiae* strain BY4741 (*MAT* **a** *his3*Δ*1 leu2*Δ*0 met15*Δ*0 ura3*Δ*0*) and TET::DED1 yeast strain (yTHC library, *MAT* **a** *his3*Δ*1 leu2*Δ*0 met15*Δ*0* URA3::CMV-tTA) was obtained from Open Biosystems (Huntsville, AL, USA). The plasmid pESC-HIS-Gal-His33/Gal-DI-72 expressing *Cucumber necrosis virus* (CNV) 6×His-tagged p33 and the TBSV DI-72 repRNA was described earlier [30]. The CNV p33 protein has very high sequence identity with the closely related TBSV p33, but the CNV p33 is expressed better and more active in yeast than TBSV p33. Recombinant yeast Tdh2p protein was produced in *E. coli* as GST fusion using plasmid pGEX-TDH2, described earlier [50]. Recombinant Ded1p helicase proteins were produced in E. coli as maltose binding protein (MBP) fusions [48]. The expression plasmid pMal-DED1 was prepared by PCR using primers #3956 (CCAGCTGCAGTCACCACCAAGAAGAGTTG)/ #3957(CCAGGAATTCATGGCTGAACTGAGCGAACAAG) and the yeast genomic DNA as a template. The plasmids pMal-Dx, expressing different Ded1p mutants, were prepared by PCR using #3956/ #3957 primers and plasmids containing mutated *DED1* sequences [41], [53]. The PCR products obtained from *DED1* wt and mutant sequences were digested with *EcoR*I and *Pst*I and inserted between *EcoR*I and *Pst*I sites in pMalc-2× (New England Biolab).

To express recombinant FHV protein A in yeast, we generated plasmid pGAD/Cup/FHV/protein A/C-term/HA/FLAG. The following sequence was fused downstream to the full length FHV protein A coding region by repeated PCR and cloning steps: GGAGGTTCCGGGGGCTCTGGAGGTTACCCATACGATGTTCCAGATTACGCTTCCGG-GGATTACAAGGACGATGACGATAAGTAACTCGAGCTCC (coding for G G S G G S G G Y P Y D V P D Y A S G D Y K D D D D K amino acids). The PCR product was amplified with oligos #3629 (CCGATCATGACTCTAAAAGTTATTCTTGGAG) and #3716 (GGAGCTCGAGTTACTTATCGTCATCGTC) followed by digestion with *Pag*I and *Xho*I. It was cloned into *Nco*I and *Xho*I digested pCupHis92 [70].

### Recombinant protein purification from *E. coli*

Expression and purification of the recombinant MBP-tagged host proteins and the MBP-tagged TBSV p33 and p92 replication proteins and Ded1p helicase from *E. coli* were carried out as described earlier with modifications [71]. Briefly, the expression plasmids were transformed separately into *E. coli* strain BL21(DE3) CodonPlus. Protein expression was induced using isopropyl β-D-thiogalactopyranoside (IPTG) for 8 h at 23°C in the case of host proteins and at 16°C in the case of p33 and p92, then the cells were collected by centrifugation (5,000 rpm for 5 min). The cells were suspended and sonicated in MBP column buffer containing 30 mM HEPES-KOH pH 7.4, 25 mM NaCl, 1 mM EDTA, 10 mM β-mercaptoethanol. The extract was then centrifuged at 16,000 g for 10 min, followed by incubation with amylose resin (NEB) for 15 min at 4°C. After washing the resin 2 times with the column buffer, the proteins were eluted with column buffer containing 0.18% (V/W) maltose. Purification of GST-tagged TDH2 (pGEX-TDH2) [50] was carried out using glutathione resin and eluted with 10 mM glutathione, 10 mM ß-mercaptoethanol in the column buffer following the same protocol as MBP-proteins. Eluted proteins were aliquoted for storage at −80°C. Protein fractions used for the replication assays were at least 95% pure, as determined by SDS-PAGE (not shown). We have previously shown that our Ded1p preparation has helicase activity on short RNA/DNA duplexes *in vitro* [48].

### RNA probes and RNA competitors used for RNA–protein interactions

The ^32^P-labeled or unlabeled full-length DI-72 (+) and (−)RNAs and the four separate regions (RI-IV), were generated as described [57]. Transcripts for replicase or CFE replication assays were purified as described earlier [57]. The amounts of transcripts were quantified by UV spectrophotometer (Beckman). To obtain full-length FHV-derived DI-634 RNA [72], we used primers #3842 (GTAATACGACTCACTATAGTAAACAATTCCAAGTTCCAAAATGG) and #3509 (ACCTTAGTCTGTTGACTTAAACTGG) or #3519 (GTAAACAATTCCAAGTTCC) and #3527 (GTAATACGACTCACTATAGGGAACCTTAGTCTGTTGACTTAAAC) for (+) or (−)DI-634 RNAs, respectively, and pDI634 [72] as a template in PCR reactions. The RNA transcripts were synthesized on the PCR templates using T7-based transcription [47].

### *In vitro* TBSV replication assay in cell-free yeast extract

CFEs from BY4741 or TET::DED1 strains capable of supporting TBSV replication *in vitro* were prepared as described earlier [23], [40]. Briefly, the *in vitro* TBSV replication assays were performed in 20-µl total volume containing 2 µl of CFE, 0.15 µg DI-72 (+)repRNA transcript, 200 ng purified MBP-p33, 200 ng purified MBP-p92^pol^ (both recombinant proteins were purified from *E. coli*), 30 mM HEPES-KOH, pH 7.4, 150 mM potassium acetate, 5 mM magnesium acetate, 0.13 M sorbitol, 0.4 µl actinomycin D (5 mg/ml), 2 µl of 150 mM creatine phosphate, 0.2 µl of 10 mg/ml creatine kinase, 0.2 µl of RNase inhibitor, 0.2 µl of 1 M dithiothreitol (DTT), 2 µl of 10 mM ATP, CTP, and GTP and 0.25 mM UTP and 0.1 µl of [^32^P]UTP [23], [40]. Host proteins were added in different amounts as indicated in the Figure legends. The reaction was performed as described [23], [40]. The newly synthesized ^32^P-labeled RNA products were separated by electrophoresis in a 5% polyacrylamide gel (PAGE) containing 0.5× Tris-borate-EDTA (TBE) buffer with 8 M urea. To detect the double-stranded RNA (dsRNA) in the cell-free replication assay, the ^32^P-labeled RNA samples were divided into two aliquotes: one half was loaded onto the gel without heat treatment in the presence of 25% formamide, while the other half was heat denatured at 85°C for 5 min in the presence of 50% formamide [27].

Fractionation of the whole cell extract was done according to [23], [40]. The total extract was centrifuged at 21,000× g at 4°C for 10 min to separate the “soluble” (supernatant) and “membrane” (pellet) fraction. The pellet was re-suspended and washed with buffer A (30 mM HEPES-KOH pH 7.4, 150 mM potassium acetate, and 5 mM magnesium acetate) followed by centrifugation at 21,000× g at 4°C for 10 min and re-suspension of the pellet in buffer A. *In vitro* TBSV replication in the fractions was performed as described [23], [40].

### Tombusvirus replicase purification from yeast and *in vitro* RdRp assay

Yeast strains (BY4741 and TET::DED1) were transformed with plasmids pGBK-HisFlagp33 (or pGBKHisp33 as a control), pGAD-HisFlagp92 (or pGAD-Hisp92 as a control) and pYC-DI72. The 6×His-Flag double-tagged HF-p33 and p92 were expressed from the *ADH1* promoter and DI-72 repRNA was under the *Gal1* promoter. Transformed yeast were pre-grown on SC-ULH^−^ media containing 2% glucose at 29°C. After centrifugation at 2,000 rpm for 3 min and washing pellet with selective media containing 2% galactose and 1 mg/ml Doxycycline, yeast were grown for 24 hours in SC-ULH^−^ media containing 2% galactose at 23°C. The replicase purification was done according to a previously described procedure [24] with the following modification. Briefly, 200 mg of yeast cells were re-suspended and homogenized in TG buffer [50 mM Tris–HCl [pH 7.5], 10% glycerol, 15 mM MgCl_2_, 10 mM KCl, 0.5 M NaCl, , and 1% [V/V] yeast protease inhibitor cocktail (Ypic)] by glass beads using FastPrep Homogenizer (MP Biomedicals). The membrane fraction containing the viral replicase complex was solubilized with 1 ml TG buffer containing 1% Triton X-100, 1% [V/V] Ypic as described [14], [15]. After affinity purification of HF-p33 on anti-FLAG M2-agarose affinity resin (Sigma), the resin-bound replicase complex was eluted in 100 µl elution buffer [50 mM Tris–HCl [pH 7.5], 10% glycerol, 15 mM MgCl_2_, 10 mM KCl, 50 mM NaCl, 1% Triton X-100, and 0.15 mg/ml Flag peptide (sigma)].

*In vitro* RdRp activity assay was performed by using DI-72(−) or (+) RNA template transcribed *in vitro* by T7 transcription [14]. To measure the effect of host proteins in the replicase assay with DI72(−) RNA containing short double-stranded region at the 3′-end, a heat denatured RNA transcript (94°C for 2 min) was annealed with a 21-nt oligodeoxynucleotide (#20) (in 1∶10 molar ratio) complementary to the 3′ end of DI-72(−)RNA in STE buffer (10 mM TRIS, pH 8.0, 1 mM EDTA, and 100 mM NaCl) and then slowly (in 30 min) cooled them down to 25°C (Panavas and Nagy, 2005). RNase ONE digestion to remove single-stranded ^32^P-labeled RNA was performed at 37°C for 30 min in a 1× RNase ONE buffer containing 0.1 µl of RNase ONE (Promega).

### FHV replicase purification from yeast and *in vitro* replicase assay

To obtain FHV replicase preparation, BY4741 yeast strain was transformed with plasmid pGAD/Cup/FHV/proteinA/C-term/HA/FLAG and pESC-His-GAL1::FHVRNA1framshift. After selection of transformed yeast on SC-LH^−^ plates, yeast were pre-grown overnight in selective media containing 2% glucose at 29°C. After centrifugation at 2,000 rpm for 3 min and washing pellet with selective media containing 2% galactose, yeast were grown 36 hours at 29°C in SC-LH^−^ media containing 2% galactose and 50 µM CuSO_4_ to induce FHV RNA replication. Affinity-purification was done similarly as for tombusvirus, except for using different buffers: homogenization buffer consisted of 50 mM Tris–HCl [pH 8.0], 0.4 M sorbitol, 5 mM MgCl_2_, 50 mM KCl, 0.03% β -mercaptoethanol and 1% [V/V] yeast protease inhibitor cocktail (Ypic)] and solubilization (washing) and elution buffers contained 50 mM Tris–HCl [pH 8.0], 0.4 M sorbitol, 5 mM MgCl_2_, 50 mM KCl, 0.5 M NaCl, 0.03% β-mercaptoethanol, 1% Triton X-100 and 1% [V/V] yeast protease inhibitor cocktail (Ypic)]. Elution buffer also contained 0.15 mg/ml Flag peptide (Sigma)].

### Analysis of protein–protein interactions using the split-ubiquitin assay

The split-ubiquitin assay was based on the Dualmembrane kit3 (Dualsystems). The bait construct, pGAD-BT2-N-His33, expressing the CNV p33 replication protein has been described earlier [26]. The prey constructs were made by PCR amplification of individual genes using gene specific primers: #3957 (CCAGCTGCAGTCACCACCAAGAAGAGTTG) / #4602 (CCAGCCATGGCCACCAAGAAGAGTTG) followed by digestion with *EcoR1* and *Nco1* for *DED1* and #4312(CCAGGGATCCATGACTTACGGTGGTAGAG) / #4603 (CCAGCCATGGATAGTTTGAACGACCTC), #4318 (CCAGGGATCCATGAGTCGCTACGATAGCCG) / #4604 (CCAGCCATGGGCTCCACCCTCTTCTGCTC) followed by digestion with *BamH1* and *Nco1* in the case of RH20 gene, respectively. Digested PCR products were fused to NubG at either the 5′- or 3′- termini (NubG-x and x-NubG) by cloning into pPRN-N-RE or pPRN-C-RE vectors [26], respectively, using the same enzymes. Yeast strain NMY51 was co-transformed with pGAD-BT2-N-His33 and pPR-N-RE or one of the prey constructs carrying the cDNA for a given helicase and plated onto Trp^−^/Leu^−^ synthetic minimal medium plates. Transformed colonies were picked with a loop, re-suspended in water, and streaked onto TLHA^−^ (Trp^−^/Leu^−^/His^−^/Ade^−^) plates to test for p33–helicase protein interactions as described [26].

### Protein co-purification with the viral replicase

*S. cerevisiae* strain DED1::6×HA-hphNT1 was generated by homologous recombination using strain BY4741. PCR was performed using plasmid pYM-16 (EUROSCARF) [73] as template and primers #2493 (GCAGAAAACGAAGAATCCTCACCCTAGTTTGTCTGAAATCAATCGATGAATTCGAGCTCG) / #2494 (GGCTGGGGTAACAGCGGTGGTTCAAACAACTCTTCTTGGTGGCGTACGCTGCAGGTCGAC). The PCR products were transformed to BY4741 and recombinant yeast colonies were selected in YPD plates supplemented with hygromycin. Recombinant yeast strains were transformed with plasmids pGBK-HisFlagp33, pGAD-HisFlagp92 and pYC-DI72 [24]. 6×His/Flag-tagged HF-p33 and HF-p92 were expressed from *ADH1* promoter and DI-72 transcript was under *GAL1* promoter. After selection of transformed yeast on SC-ULH^−^ plates, yeast were pre-grown overnight in selective media containing 2% glucose at 29°C. After centrifugation at 2,000 rpm for 3 min and washing the pellet with selective media containing 2% galactose, yeast were grown for 36 hours in SC-ULH^−^ media containing 2% galactose at 23°C. 200 µl of pelleted yeast were used to affinity-purify HF-p33 and HF-p92 with anti-FLAG M2 agarose as described previously (see also Text S1) [14], [15]. HF-p33 and HF-p92 were detected with anti-His_6_ antibody (1/5,000 dilution) and AP-conjugated anti-mouse antibody (1/5,000). DED1-6×HA protein was detected with anti-HA antibody from rabbit (Bethyl; 1/10,000 dilution) and AP-conjugated anti-rabbit (1/10,000) followed by NBT-BCIP detection.

## Supporting Information

Figure S1 Reduced TBSV replication in CFE prepared from Ded1-depleted yeast. CFEs were prepared from TET::DED1 yeast strain cultured in the absence of doxycycline (i.e., Ded1p is expressed) or in its presence (10 mg/ml) (i.e., Ded1p is depleted). The CFEs were programmed with recombinant p33/p92 and DI-72(+) repRNA as described in REF 21. Note that the dsRNA product represents the annealed (−)RNA and the (+)RNA, while the ssRNA products represents the newly made (+)RNA products. The CFEs contained comparable amounts of host proteins (not shown).
(EPS)

Figure S2 Co-purification of the p33 replication protein with Ded1p from yeast. (A) 6×His/ FLAG-tagged Ded1p was purified from yeast extracts containing 6×His/FLAG -Ded1p (lane 1) or not containing 6×His/FLAG-tagged Ded1p (lane 2) using a FLAG-affinity column Top panel: Western blot analysis of p33 tagged with 6×HA with anti-HA antibody in the purified Ded1p preparations. Bottom panel: Western blot analysis of 6×His/FLAG-tagged Ded1p with anti-His antibody. Each experiment was repeated three times. (B) Co-purification of tombusvirus replicase activity with Ded1p from yeast. FLAG-tagged Ded1p was purified from yeast expressing FLAG-Ded1p (lanes 2–4) or from yeast not expressing FLAG-Ded1p (lane 5) using a FLAG-affinity column. Yeast also co-expressed p33/p92/DI-72 repRNA as indicated by “+”. External DI-72(−)RNA template was given to samples as shown. Representative denaturing gel of ^32^P-labeled RNA products synthesized *in vitro* by the purified FLAG-Ded1p preparation is shown. Lane 1 is a size-control [621 nt DI-72(+) RNA].
(EPS)

Figure S3 Ded1p mutants with ATPase activity promote plus-strand synthesis by the affinity-purified tombusvirus replicase. (A) Scheme of the tombusvirus replicase assay. Yeast with depleted Ded1p co-expressing p33 and p92^pol^ replication proteins and DI-72 (+)repRNA were used to affinity-purify the RNA-free tombusvirus replicase. The *in vitro* assays were programmed with DI-72 (−)repRNA, and they also contained purified recombinant Ded1p. (B) Representative denaturing gel of ^32^P-labeled RNA products synthesized by the purified tombusvirus replicase *in vitro* in the presence of 1.0 µg of purified recombinant Ded1p. The level of complementary RNA synthesis producing “repRNA” (marked as “FL”, the full-length product, made via initiation from the 3′-terminal promoter) in each sample was compared to that of the replicase activity obtained in the absence of added recombinant protein. Note that this replicase preparation also synthesizes internal initiation products (“ii”) and 3′-terminal extension products (“3′TEX”). RNAse One digestion was used to confirm the replicase products: the *de novo* products are insensitive, while the 3′TEX product changes migration after RNase treatment (marked with an open arrowhead in lanes 2 and 4). Each experiment was repeated three times. (C) The ATPase activities of the Ded1p mutants based on REF 38. (D) Representative denaturing gel of ^32^P-labeled RNA products synthesized by the purified tombusvirus replicase *in vitro* in the presence of 1.0 µg of purified recombinant Ded1p mutants. See Panel B for further details.
(EPS)

Figure S4 Ded1p helicase does not promote minus-strand synthesis by the affinity-purified tombusvirus and FHV replicases. (A) Scheme of the procedure. Yeast with depleted Ded1p co-expressing p33 and p92^pol^ replication proteins and DI-72 (+)repRNA were used to affinity-purify the RNA-free tombusvirus replicase. (B) Representative denaturing gel of ^32^P-labeled RNA products synthesized by the purified tombusvirus replicase *in vitro* in the presence of 0.5 µg of purified recombinant Ded1p or the D1 mutant is shown. The *in vitro* assays were programmed with DI-72 (+)repRNA, and they also contained purified recombinant Ded1p (lane 3) or the D1 mutant (lane 4) in addition to ATP/CTP/GTP and ^32^P-UTP. (C) Representative denaturing gel of ^32^P-labeled RNA products synthesized by the purified FHV replicase *in vitro* in the presence of 1.0 µg of purified recombinant Ded1p or D1 mutant on the TBSV DI-72 (+)RNA template. (D) Representative denaturing gel of ^32^P-labeled RNA products synthesized by the purified FHV replicase *in vitro* in the presence of 1.0 µg of purified recombinant Ded1p or D1 mutant on the FHV DI-634 (+)RNA template.
(EPS)

Figure S5 Ded1p binds to various regions of TBSV (+)RNA *in vitro*. (A) *In vitro* binding assay with purified Ded1p using ^32^P-labeled ssRNA templates representing the four regions of DI-72(+) RNA. The assay contained ∼0.1 pmol labeled RNA plus 1.0 µg of purified recombinant Ded1p. The unlabeled competitor was DI-72(+) RNA (3 pmol). The free or Ded1p-bound ssRNA was separated on nondenaturing 5% acrylamide gels.
(EPS)

Figure S6 Interaction between Ded1p and the TBSV replication proteins. (A) Split ubiquitin assay was used to test binding between p33 and the shown full-length proteins. The bait p33 was co-expressed with the prey proteins in yeast. Ssa1p (HSP70 chaperone), and the empty prey vector (NubG) were used as positive and negative controls, respectively. The image shows 10-fold serial dilutions of yeast cultures. (B) *In vitro* pull-down assay of GST-His-tagged Ded1p with MBP-p33 or MBP-p92 using amylose resin. Top panel: Western blot analysis with anti-His antibody of GST-His-tagged Ded1p co-purified with MBP-p33 and MBP-p92. MBP was used as a negative control. Bottom panel: Coomasie stained SDS-PAGE gel, showing quality and quantity of purified MBP, MBP-p33 and MBP-p92 proteins. (C) *In vitro* reverse pull-down assay of MBP-p33 or MBP-p92 with GST-His-tagged Ded1p using glutathione resin. Western blot analysis with anti-MBP antibody of MBP-p33 and MBP-p92 co-purified with GST-His-tagged Ded1p. GST-His was used as a negative control. Bottom panel: Coomasie stained SDS-PAGE gel, showing quality and quantity of purified proteins GST-HisDED1 and GST-His.
(EPS)

Figure S7 Comparison of the amino acid sequence of the yeast Ded1p (top) and the *Arabidopsis* RH20 helicases.
(PDF)

Figure S8 Ded1p binds to FHV RNA *in vitro*. (A) *In vitro* binding assay with purified Ded1p using ^32^P-labeled FHV and TBSV RNA templates representing (+) and (−)RNAs. The assay contained ∼0.1 pmol labeled RNA plus 0.4 µg (lanes 3, 6, 8) or 1.0 µg (lanes 2, 5, 7) of purified recombinant Ded1p. The free or Ded1p-bound ssRNAs were separated on nondenaturing 5% acrylamide gels. (B) *In vitro* pull-down of GST-His-tagged Ded1p or GST-His with MBP-protA of FHV using amylose resin. Top panel: Western blot analysis with anti-His antibody of GST-His-tagged Ded1p co-purified with MBP-protA. MBP and GST-His were used as negative controls. Bottom panel: Coomasie stained SDS-PAGE gel, showing quality and quantity of purified MBP, and MBP-protA proteins.
(EPS)

Text S1 Materials and Methods used in the supplementary experiments.
(DOC)
