# Supplementary material for: A Co-Opted DEAD-Box RNA Helicase Enhances Tombusvirus Plus-Strand Synthesis
Source: PLoS Pathog. 2012 Feb 16;8(2):e1002537. doi: 10.1371/journal.ppat.1002537 (PMC3280988; doi:10.1371/journal.ppat.1002537)
Supplement: Figure S7 — Comparison of the amino acid sequence of the yeast Ded1p (top) and the Arabidopsis RH20 helicases. (PDF) [file ppat.1002537.s007.pdf]

```
VDASGKDVPEPITEFTSPPLDGLLLENIKLARFTKPTPVQKYSVPIVANGRDLMACAQTG  189
+   GKD+P+P+  F        +LE +K A FT+PTP+Q    P+   GRDL+  A+TG
ITVEGKDIPKPVKSFRDVGFPDYVLEEVKKAGFTEPTPIQSQGWPMAMKGRDLIGIAETG  146

SGKTGGFLFPVLSESFKTGPSPQPESQGSFYQRKAYPTAVIMAPTRELATQIFDEAKKFT  249
SGKT  +L P +        + QP            P  +++APTRELA QI  EA KF
SGKTLSYLLPAIVHV-----NAQP-----MLAHGDGPIVLVLAPTRELAVQIQQEASKFG  196

YRSWVKACVVYGGSPIGNQLREIERGCDLLVATPGRLNDLLERGKISLANVKYLVLDEAD  309
S +K   +YGG P G Q+R++++G ++++ATPGRL D++E    +L  V YLVLDEAD
SSSKIKTTCIYGGVPKGPQVRDLQKGVEIVIATPGRLIDMMESNNTNLRRVTYLVLDEAD  256

RMLDMGFEPQIRHIVEDCDMTPVGERQTLMFSATFPADIQHLARDFLSDYIFLSVGRVG-  368
RMLDMGF+PQIR IV    + P  +RQTL +SAT+P +++ L++ FL +   + +G
RMLDMGFDPQIRKIV--SHIRP--DRQTLYWSATWPKEVEQLSKKFLYNPYKVIIGSSDL  312

STSENITQKVLYVENQDKKSALLDLLSASTDG-LTLIFVETKRMADQLTDFLIMQNFRAT  427
  +  I Q V  +    K + L+ LL    DG   L+F++TK+  DQ+T  L M  + A
KANRAIRQIVDVISESQKYNKLVKLLEDIMDGSRILVFLDTKKGCDQITRQLRMDGWPAL  372

AIHGDRTQSERERALAAFRSGAATLLVATAVAARGLDIPNVTHVINYDLPSDVDDYVHRI  487
+IHGD++Q+ER+  L+ FRSG + ++ AT VAARGLD+ +V +VINYD P  ++DYVHRI
SIHGDKSQAERDWVLSEFRSGKSPIMTATDVAARGLDVKDVKYVINYDFPGSLEDYVHRI  432

GRTGRAGNTGLATAFFNSENSNIVKGLHEILTEANQEV  525
GRTGRAG  G A  FF   N+   K L  IL EA Q+V
GRTGRAGAKGTAYTFFTVANARFAKELTNILQEAGQKV  470
```
