# Supplementary material for: A Co-Opted DEAD-Box RNA Helicase Enhances Tombusvirus Plus-Strand Synthesis
Source: PLoS Pathog. 2012 Feb 16;8(2):e1002537. doi: 10.1371/journal.ppat.1002537 (PMC3280988; doi:10.1371/journal.ppat.1002537)
Supplement: Text S1 — Materials and Methods used in the supplementary experiments. (DOC) [file ppat.1002537.s009.doc]

**Text S1. Materials and Methods used in the supplementary experiments.**

# In vitro pull-down assay. pGST-His-DED1 was obtained as follows: *DED1* sequence was amplified using pMAL-DED1 as a template and primers #3957 and #4309 CCAGCTCGAGTCACCACCAAGAAGAGTTG. The obtained PCR product was digested with *Xho*1/*EcoR*1 and inserted into pGEX-His-RE [1] plasmid which was digested with the same enzymes. Product from the empty pGEX-His-RE plasmid was used as GST control. Purification of GST-tagged proteins were carried out using glutathione resin and eluted with 10 mM glutathione, 10 mM ß-mercaptoethanol in the column buffer following the same protocol as MBP-proteins [1].

# MBP-binding columns were used to bind the MBP-p33 or MBP-p92 (described previously) [2] or MBP-FHVprtA. After addition of the *E. coli* sonicated extracts containing MBP-tagged viral proteins and incubation for 20 min at 4°C with continuous mixing, the columns were washed three times with cold column buffer prior to loading of purified GST-tagged Ded1p or GST (negative control). After purified GST-tagged proteins (100 μg) being loaded onto columns, containing pre-bound MBP-tagged viral proteins, we incubated the mixture for 30 min at 4°C with mixing. The columns were then washed three times with cold column buffer, and the bound protein complexes were eluted with column buffer containing 0.18% (V/W) maltose. The presence of GST-His-tagged proteins in the eluate was analyzed by sodium dodecyl sulfate-polyacrylamide gel electrophoresis (SDS-PAGE), followed by Western blotting with an anti-His antibody. The amount of MBP-tagged viral proteins in the eluate was visualized by Coomassie blue staining of the SDS-PAGE gels.

**In vitro reverse pull-down assay.** GST-binding columns were used to bind the GST-tagged Ded1 or GST (negative control). After addition of the sonicated E. coli extracts containing GST-tagged Ded1 or GST and incubation for 20 min at 4°C with mixing, the columns were washed three times with cold column buffer prior to loading of purified MBP-tagged viral proteins p33 or p92 (50 μg). After incubation for 30 min at 4°C with mixing, the columns were washed three times with cold column buffer, and the bound protein complexes were eluted with column buffer supplemented with 10 mM glutathione. The presence of MBP-tagged viral proteins in the eluate was analyzed by SDS-PAGE, followed by Western blotting with an anti-MBP antibody (NEB). The amount of GST-tagged Ded1 or GST in the eluate was visualized by Coomassie blue staining of the SDS-PAGE gels.

**Co-purification of p33, p92 and replicase with Ded1 from yeast.** Yeast over-expression plasmid pYC-HisFlag-DED1 was prepared as follows. *DED1* sequence was amplified using primers #3957/#4309. The obtained PCR-product was digested with *EcoR*1 and *Xho1* and inserted into pYC-HisFlag vector, which was digested with the same enzymes.

# pESC-His/HA/CNV33, expressing CNV p33 with an N-terminal HA-tag, was generated using PCR from pHisGBKCupHisCNV33 [3,4] using oligos #3995 (CGGCCATGGATTACCCATACGATGTTCCAGATTACGCTGATACCATCAAGAGGATGCTG) and #1403 (GCCGCTCGAGCTATTTCACACCAAGGGACTCA). The PCR product was digested with NcoI and XhoI and ligated into NcoI and XhoI digested pESC-His/Cup/HF. pGAD/HA/CNV92, expressing CNV p92 with an N-terminal HA-tag, was generated by PCR from pGADCupHis92 [3,4] using oligos 3995 and 952 (CCCGCTCGAGTCATGCTACGGCGGAGTCAAGGA). The PCR product was digested with NcoI and XhoI and ligated into NcoI and XhoI digested pGAD/Cup/HisCNV92.

Yeast parental strain BY4741 was transformed with pYC-HisFlag-DED1 plasmid and pESC-His/HA/CNV33, pGAD/HA/CNV92 plasmids in order to test co-purification of replicase proteins with Ded1. Yeast transformed with the empty pYC-HisFlag vector was used as a control. Transformed yeast were selected on SC-ULH^-^ plates, then pre-grown for 36 hours in selective media containing 2% glucose at 29°C. After centrifugation at 2,000 rpm for 3 min and washing the pellet with selective media containing 2% galactose, yeast were grown for 36 hours in SC-ULH^-^ media containing 2% galactose and Cu^2+^ at 29°C. Co-purification was done according to a previously described procedure with the following modification: Briefly, 200 mg of yeast cells were re-suspended and homogenized in TG buffer [50 mM Tris–HCl [pH 7.5], 10% glycerol, 15 mM MgCl_2_, 10 mM KCl, 0.5 M NaCl, and 1% [V/V] yeast protease inhibitor cocktail (Ypic)] by glass beads using FastPrep Homogenizer (MP Biomedicals). The yeast cell lysate was cleared by centrifugation at 500g for 5 min at 4°C to remove unbroken cells and debris. The membrane fraction containing the viral replicase complex was collected by centrifugation at 38,000 g for 15 min at 4°C and then solubilized with 1 ml TG buffer containing 1% NP-40, 5% SB3-10, 1% [V/V] Ypic via gentle rotation for 3 h at 4°C. The solubilized membrane fraction was centrifuged at 38,000 g for 15 min at 4°C and the supernatant was incubated with 25 μl anti-FLAG M2-agarose affinity resin (Sigma) pre-equilibrated with 0.7 ml TG buffer overnight by gentle rotation at 4°C. Then we washed the resin for 3 times with TG buffer containing 0.1% NP-40 and the resin-bound replicase complex was eluted in 80 μl of SDS-PAGE loading buffer.

Yeast parental strain BY4741 was transformed with pYC-HisFlag-DED1 plasmid and pGBK-Hisp33(ADH)-DI72(Gal), pGAD-Hisp92(ADH). As controls we used 1) empty pYC-HisFlag vector co-transformed with pGBK-Hisp33(ADH)-DI72(Gal), pGAD-Hisp92(ADH); and 2) pYC-HisFlag-DED1 plasmid co-transformed with pGBK and pGAD vectors. Transformed yeast were selected on SC-ULH^-^ plates, then cultured for 48 hours in selective media containing 2% galactose at 29°C. Co-purification was done according to a previously described procedure with some modification [REF 12, 13 in the main text]. Briefly, 200 mg of yeast cells were re-suspended and homogenized in TG buffer [50 mM Tris–HCl [pH 7.5], 10% glycerol, 15 mM MgCl_2_, 10 mM KCl, 0.5 M NaCl, and 1% [V/V] yeast protease inhibitor cocktail (Ypic)] by glass beads using FastPrep Homogenizer (MP Biomedicals). The yeast cell lysate was cleared by centrifugation at 500g for 5 min at 4°C to remove unbroken cells and debris. The membrane fraction containing the tombusviral replicase complex was separated from soluble fraction by centrifugation at 38,000 g for 15 min at 4°C. The membrane fraction was then solubilized with 1 ml TG buffer containing 1% NP-40, 5% SB3-10, 1% [V/V] Ypic via gentle rotation for 3 h min at 4°C. The solubilized membrane fraction was centrifuged at 38,000 g for 15 min at 4°C and the supernatant was added to soluble fraction and incubated with 50 μl anti-FLAG M2-agarose affinity resin (Sigma) pre-equilibrated with 0.7 ml TG buffer overnight with gentle rotation at 4°C. Then we washed the resin 3 times with TG buffer containing 0.1% NP-40, the resin-bound replicase complex was eluted in 100 μl elution buffer [50 mM Tris–HCl [pH 7.5], 10% glycerol, 15 mM MgCl_2_, 10 mM KCl, 50 mM NaCl, 0.1% NP-40, and 0.15 mg/ml FLAG peptide (Sigma)]. From the 100 μl of purified samples 20 μl were taken into the in vitro RdRp reaction, using DI-72(-) as a substrate and containing 10 mM ATP, CTP, and GTP and 0.3 μl of [^32^P]UTP.

References

1. Barajas D, Li Z, Nagy PD (2009) The Nedd4-type Rsp5p ubiquitin ligase inhibits tombusvirus replication by regulating degradation of the p92 replication protein and decreasing the activity of the tombusvirus replicase. J Virol 83: 11751-11764.

2. Rajendran KS, Pogany J, Nagy PD (2002) Comparison of turnip crinkle virus RNA-dependent RNA polymerase preparations expressed in Escherichia coli or derived from infected plants. J Virol 76: 1707-1717.

3. Serviene E, Jiang Y, Cheng CP, Baker J, Nagy PD (2006) Screening of the yeast yTHC collection identifies essential host factors affecting tombusvirus RNA recombination. J Virol 80: 1231-1241.

4. Jiang Y, Serviene E, Gal J, Panavas T, Nagy PD (2006) Identification of essential host factors affecting tombusvirus RNA replication based on the yeast Tet promoters Hughes Collection. J Virol 80: 7394-7404.
